# Effects of root pruning on the physicochemical properties and microbial activities of poplar rhizosphere soil

**DOI:** 10.1371/journal.pone.0187685

**Published:** 2017-11-08

**Authors:** Da-Wei Jing, Fang-Chun Liu, Ming-You Wang, Hai-Lin Ma, Zhen-Yu Du, Bing-Yao Ma, Yu-Feng Dong

**Affiliations:** 1 College of Resources Environment and Planning, Dezhou University, Dezhou, China; 2 Institute of Resource and Environment, Shandong Academy of Forestry, Jinan, China; 3 College of Ecology and Garden Architecture, Dezhou University, Dezhou, China; RMIT University, AUSTRALIA

## Abstract

This study aimed to determine the effects of root pruning on the physicochemical characteristics and microbial activities of poplar rhizosphere soil. The root systems of 5-year-old poplar (*Populus×euramericana cv*. ‘Neva’) trees were manually pruned at 6, 8, or 10 times diameter at breast height (DBH) from the trunk (severe, moderate, and light, respectively) along both inter-row sides. Moderate root pruning significantly increased the concentrations of amino acids, organic acids, and total sugars in the root exudates and decreased the pH of rhizosphere soil. This treatment also increased the contents of available nitrogen, phosphorus, potassium, and total organic carbon as well as high-, medium-, and low-activity organic carbon in rhizosphere soil. Moreover, moderate pruning increased the contents of microbial biomass carbon and nitrogen, and enhanced basal respiration, in addition to decreasing the metabolic quotients in rhizosphere soil by 8.9%, 5.0%, and 11.4% compared with control, light, and severe root pruning treatments, respectively. Moderate pruning increased the growth rates of DBH, tree height, and volume to the highest levels. Furthermore, these indices were not significantly different between the light root pruning and control groups, but varied significantly between severe and moderate root-pruning treatments. Thus, root pruning, depending on the distance from the trunk, significantly influences the physicochemical properties and microbial activities in poplar rhizosphere soil.

## Introduction

A root system is an absorptive and metabolic organ of a plant and displays sensitive reactions to external environmental conditions [1]. Soil conditions, such as soil nutrients, water content, and aeration status, directly influence the growth, distribution, absorption function, and metabolic function of root systems, which then affect the growth, development, and output of the aboveground parts of the plant [2]. Poplar is the main afforestation tree species of fast-growing and high-yielding plantation projects in China [3].Poplar trees are mainly planted at a density of 830–1100 trees per ha in China [3].As the poplar tree grows, the lateral roots gradually expand and inevitably intermingle with those of adjacent trees when the canopy of the poplar plantation is closed [4]. The interwoven roots slow down the growth of the poplar tree and promote the spread of pests and diseases, thereby significantly reducing the root activity [5]. Moreover, the amount of fine roots sharply decreases, suppressing the ability of the root system to absorb water and nutrients [4, 6]. Therefore, methods for improving root system activity in closed-canopy poplar plantations must be developed to promote tree growth.

Root pruning is a cultivation method used to adjust the above- and belowground plant sections and to modify the vegetative and reproductive growth processes of plants by controlling the growth of root systems [7]. Root pruning is commonly used in agricultural production to control canopy size during the vegetative growth of apple [8, 9], pear [10], and peach [11–12] trees to enable them to adapt to the dwarf habit and close planting requirements of modern fruit tree plantations. Root pruning can also mitigate premature fruit drop before harvesting [13]. Therefore, root pruning is an effective method for inhibiting vegetative growth [7]. Previous studies on winter jujube reported that root pruning stimulates the germination of a large number of fine roots from the point of incision; root exudates secreted by the resulting fine roots not only significantly increase the enzyme activities and microbial population but also enhance the effectiveness and supply capabilities of nutrients in the soil, resulting in improved soil fertility [14]. Therefore, the removal of lateral roots through root pruning in closed-canopy poplar could induce the germination of new roots and promote the absorption of nutrients by the root system to enhance the growth of these economically important trees.

The rhizosphere is the zone of soil surrounding a plant root where the biology and chemistry of the soil are significantly influenced by the living roots. This zone is approximately 1–2 mm wide, but has no distinct edge [15]. Rather, it is an area of intense biological and chemical activity influenced by compounds exuded by the root, and by microorganisms feeding on those compounds. The chemical components of the rhizosphere can differ from those of bulk soil because of root exudation, nutrient uptake, and microbial activity [16]. Furthermore, the soil microbial biomass functions as the source and sink of available nutrients in plants and is closely related to soil quality [17]. The microbial quotient (*q*MIC) refers to the percentage of microbial biomass carbon (MBC) to soil organic carbon (SOC) and is an effective indicator of the improvement or deterioration in soil quality [18]. The microbial metabolic quotient (*q*CO_2_), which is the amount of CO_2_-C produced per unit MBC, is as an eco-physiological measure of ecosystem succession or disturbance [19]. Soil microbial biomass, *q*MIC, and *q*CO_2_ are used as indicators of soil development or degradation and changes in soil quality [17, 20], and also play an important role in the nutrient storage capability of soil [21].Root pruning can affect the rhizosphere soil characteristics of poplar, but few reports are available regarding the effects of root pruning on the physicochemical properties and microbial activities of rhizosphere soil.

In this study, we investigated the effect of root pruning on root exudates, physicochemical properties and microbial activities in the rhizosphere soil and poplar growth in the plain areas of the northern China. We hypothesized that root pruning would improve the characteristics of the rhizosphere soil and lead to increased tree growth. The objective was to determine the feasibility of implementing root pruning to increase the growth of poplar at the canopy-closing stage.

## Materials and methods

### Ethics statement

This research did not involve human or other animal subjects. For plant collections, we collected the minimum number of specimens necessary to ensure that appropriate vouchers were obtained. The field studies did not involve endangered or protected species. Permission to work in a poplar plantation located in Ertun township was obtained through a cooperative agreement between Dezhou University and Dezhou Forestry Bureau.

### Site description and plant material

The experiment was conducted in a poplar plantation located in Ertun township, Dezhou city, Shandong Province, north China (36°24'N latitude, 115°45'E longitude). The area has a warm temperate zone continental monsoon climate with distinct four seasons and ample sunshine; average temperature and rainfall are 12.9°C and 547.5 mm, respectively. The main physicochemical characteristics of 0–40 cm-deep soil at the research site are detailed in Table 1. N, P_2_O_5_, and K_2_O were applied annually at rates of 205.4, 70.6, and 58.7 kg^.^ha^-1^, respectively. The fertilizers comprised urea, ammonium phosphate, and potassium chloride. In the study area, 5-year-old trees of poplar ‘I-107’ (*Populus×euramericana cv*. ‘Neva’) were planted at a spacing of 4 m between rows and 3 m within rows. The experimental trees were uniformly distributed and had an average tree height and diameter at breast height (DBH; 1.3 m) of 12.8±0.39 m and 12.3±0.32 cm, respectively. These trees were carefully managed and grown as short rotation poplar (7–8 years) mainly for pulp wood.

**Table 1:** Main physicochemical characteristics of 0–40 cm-deep soil at the research site.

Soil classification^1^	Organic matter (g/kg)	Available nitrogen (mg/kg)	Available phosphorus (mg/kg)	Available potassium (mg/kg)	pH
Entisols	11.2	34.7	14.3	86.1	8.53

^1^Soil classification was according to USDA.

### Experimental design and root-pruning treatments

The experiment involved a randomized complete block design with four treatments and three replications. Twelve plots were established, and each replication for every treatment contained a plot with 30 trees arrayed in five rows; only the innermost 12 trees, which were considered representative of the plot mean, were used for detailed measurements. Immediately after the leaves fully developed, four treatments were applied to the poplar trees at the beginning of the growing season on April 13, 2016. The treatments were as follows: (1) control, with intact root system; (2) severe, cutting the root system at six times the DBH distance from the trunk (74.1 cm); (3) moderate, cutting the root system at eight times the DBH distance from the trunk (98.8 cm); and (4) light, cutting the root system at ten times the DBH distance from the trunk (123.5 cm). The root system was cut with a sharp metal spade at different distances from the trunk on both inter-row sides of poplar trees to a depth of 30 cm. The rhizosphere soil was collected during early November 2016, which meant that the soil measurements performed at six, eight, and ten times the DBH from the trunk represented the effect of root pruning over a period of seven months. Before root pruning, the physicochemical properties and microbial activities of the rhizosphere soil collected from the points of six times, eight times and ten times the DBH from the trunk were determined (Table 2). The treated trees were managed in accordance with routine care.

**Table 2:** The physicochemical properties and microbial activities from the points of six times, eight times and ten times the DBH from the trunk of poplar before pruning (mean±SD).

Treatment	Available nutrient/(mg·kg^-1^)			pH	Total organic carbon/(g·kg^-1^)	MBC/ (mg·kg^-1^)	Basal respiration/ (mg·kg^-1^·h^-1^)	Metabolic quotient/ (mg·g^-1^·h^-1^)
	N	P	K					
**Severe**	35.27±1.26a	13.91±0.65a	91.80±10.26a	8.56±0.18a	6.45±0.12a	361.02±21.96a	0.59±0.02a	1.63±0.02a
**Moderate**	34.10±1.92a	14.59±0.86a	97.46±9.33a	8.49±0.23a	6.59±0.15a	356.45±16.87a	0.60±0.02a	1.68±0.04a
**Light**	34.52±1.70a	14.35±0.94a	80.22±9.98a	8.51±0.14a	6.50±0.19a	372.59±25.18a	0.62±0.03a	1.66±0.02a

Note: Different letters indicate significant differences among treatments at *P*<0.05 by LSD.

### Sampling of rhizosphere soil

During early November 2016, rhizosphere soil was collected using the method of Wang and Zabowski [16]. Rhizosphere soil was collected as follows: all the new roots from the incision point were removed from the soil with minimum injury to the roots. The roots were shook until loosely attached soil was removed. Meanwhile, soil that remained adhered to the root system was collected by placing the roots into a paper bag and vigorously shaking the roots. Twelve soil samples were collected from the innermost 12 trees in each plot, respectively, then mixed uniformly as a composite soil sample with three replications. After manually removing any visible roots, fauna, and organic debris, the soil samples were sieved (<2 mm) and divided into two subsamples. The first subsample was air dried at room temperature (around 20°C) and used for chemical analyses. A portion of these air-dried samples was ground with a mill and sieved (<0.25 mm) prior to analysis of root exudates, soil total organic carbon (TOC), and active organic carbon (highly-active, moderately-active, and inertly-active) analysis. The remaining portions were used to measure the soil pH. The second subsample was stored in plastic bags at 4°C for 24 h prior to the analysis of basal respiration (BR), microbial biomass carbon (MBC), and microbial biomass nitrogen (MBN).

### Analysis method

#### Soil chemical properties

Root exudates in the rhizosphere soil were determined using the method of Klein et al. [22]. Available nitrogen content in the rhizosphere soil was determined by an indophenol blue colorimetric method [23]. Available phosphorus content in the rhizosphere soil was extracted by 0.5 M NaHCO_3_ and measured using the ascorbic acid–ammonium molybdate method described by Liu and Yang [24]. Rhizosphere soil was extracted by ammonium acetate and analyzed with a flame spectrophotometer to determine available potassium [23]. pH was measured using 1:2.5 (w/v) water extraction and PHM 92 Radiometer pH Meter (Radiometer America, San Diego, USA). TOC concentration was determined by oxidation with potassium dichromate [25]. Active organic carbon was determined according to Xue et al. [26], selecting three different concentrations of KMnO_4_ solutions (33, 167, and 333 mmol^.^L^-1^) to determine different types of active organic carbon (highly-active, moderately-active, and inertly-active, respectively).

#### Soil microbial properties

Soil MBC and MBN were determined by a chloroform fumigation–extraction method [8, 27]. Briefly, 25 g of soil was pre-incubated in a humidified incubator at 25°C in the dark for 7 days. Soils treated with or without chloroform fumigation (24 h) were extracted with 50 mL of 0.5 M K_2_SO_4_ for 30 min and then filtered. Organic C and total N in the soil extract were measured by dichromate oxidation [28] and the K_2_S_2_O_8_ oxidation method [27], respectively. Microbial biomass was calculated as differences in K_2_SO_4_-extractable C or N concentration between fumigated and non-fumigated soils, divided by efficiency factors for MBC (*K*_C_ = 0.38) and MBN (*K*_N_ = 0.45), respectively. The *q*MIC was calculated as a percentage of MBC to TOC.

Basal respiration was determined by measuring CO_2_ evolution [29]. Briefly, field-moist soil (20 g of oven-dried soil) was placed in 500 -mL air-tight glass vessels and water was added to 60% of the water-holding capacity of the soil. The vessels were closed with a rubber stopper and pre-incubated at 25°C for 3 days. The vessels were then opened, closed again, and incubated for 24 h at the same temperature. CO_2_ evolved from the soil was absorbed in 10 mL of 0.2 M NaOH, and the remaining base was titrated with 0.1 M HCl. The Na_2_CO_3_ formed was precipitated by adding 2 mL of 1 M BaCl_2_. The results were expressed as microgram CO_2_-C per gram per hour. The *q*CO_2_ was calculated by dividing the hourly BR by the corresponding MBC.

#### Growth rate

Tree height and DBH were measured using the tangent method [30] and with a ruler with 0.5 mm accuracy at the beginning of the experiment (April 13, 2016) and at the end of the short rotation period (November 4, 2016). Tree volume was calculated using Eq 1:

$$V=3.14d^{2}hf\;/\;4\;\left(f=0.42\right)$$ (1)

where d and h represent DBH (cm) and tree height (m), respectively [3].

The average growth rates of DBH, tree height, and tree volume were calculated using Eqs 2–4 [31]:

$$P_{h}=\frac{\left(h_{2}-h_{1}\right)\times 200}{\left(h_{2}+h_{1}\right)\times n}$$ (2)

$$P_{d}=\frac{\left(d_{2}-d_{1}\right)\times 200}{\left(d_{2}+d_{1}\right)\times n}$$ (3)

$$P_{v}=\frac{\left(v_{2}-v_{1}\right)\times 200}{\left(v_{2}+v_{1}\right)\times n}$$ (4)

where *P*_h_, *P*_d_, and *P*_v_ represent the average growth rates (%) of tree height, DBH, and tree volume, respectively; n represents the interval years between two determinations; *h*_l_ and *h*_2_ indicate the tree height (m) before and after *n* years, respectively; *d*_1_ and *d*_2_ indicate the DBH (cm) before and after *n* years, respectively; and *v*_l_ and *v*_2_ represent the tree volume before and after *n* years (m^3^), respectively. In this study, the values of *h*_1_, *d*_1_, and *v*_1_, and those of *h*_2_, *d*_2_, and *v*_2_ were measured on April 13^th^ of 2016 and November 4^th^of 2016, respectively.

### Statistical analysis

**Fig 1:**
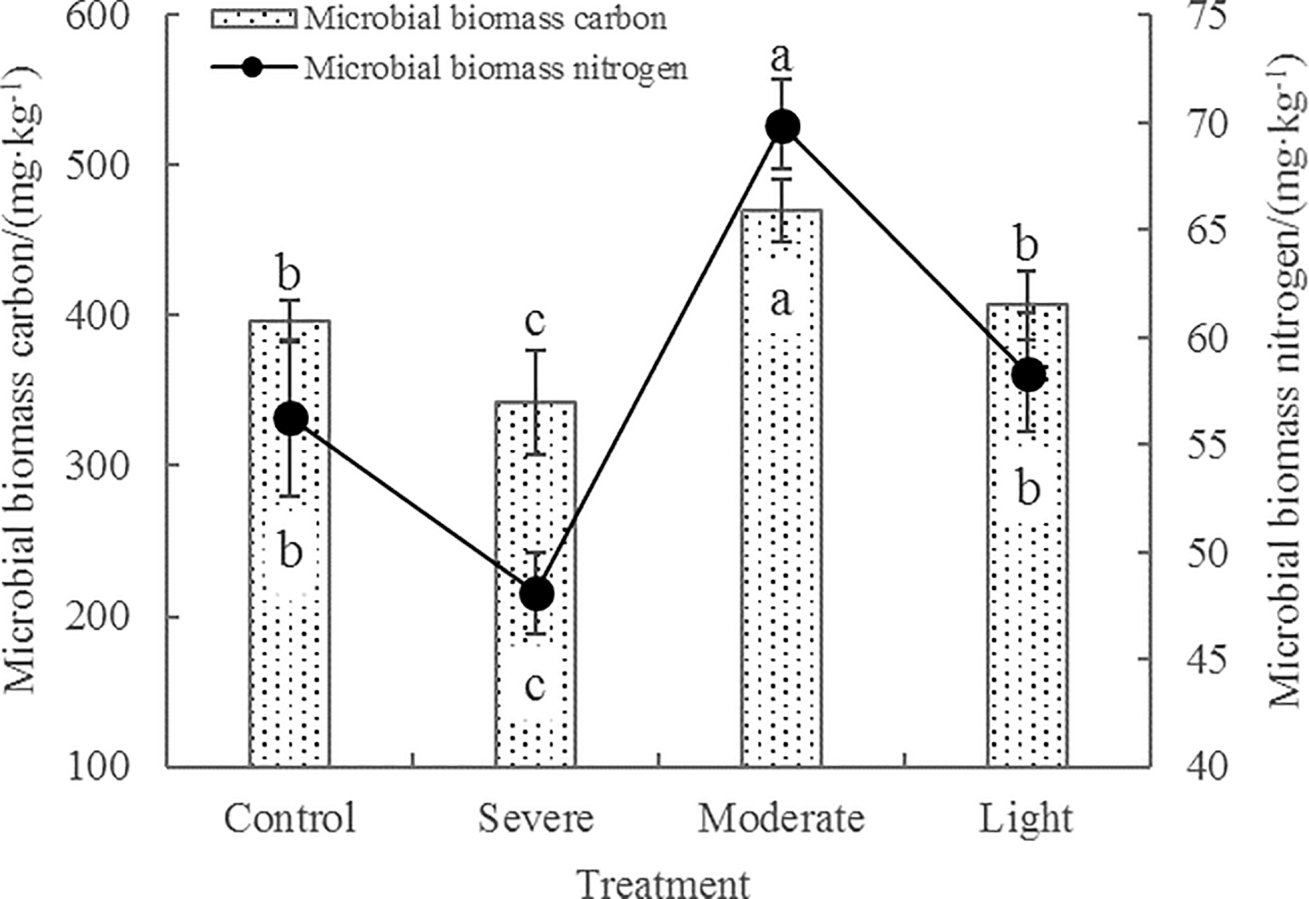
Effects of root pruning on MBC and MBN contents in the rhizosphere soil of poplar. Bars are means, and error bars are standard deviations (n = 3). Different letters indicate significant differences among treatments at *P*<0.05 by LSD.

**Fig 2:**
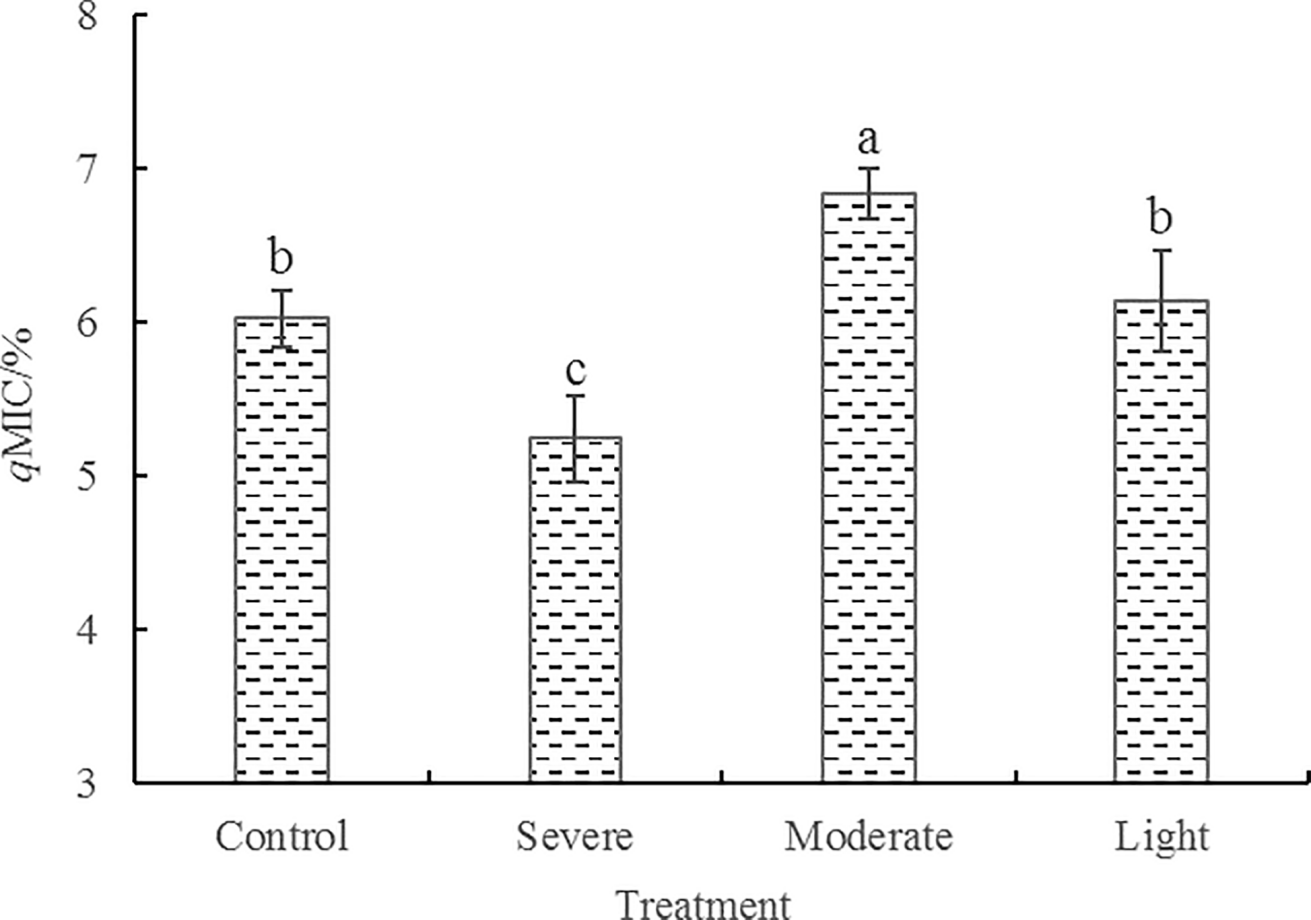
Effects of root pruning on microbial quotient (*q*MIC) in the rhizosphere soil of poplar. Bars are means, and error bars are standard deviations (n = 3). Different letters indicate significant differences among treatments at *P*<0.05 by LSD.

**Fig 3:**
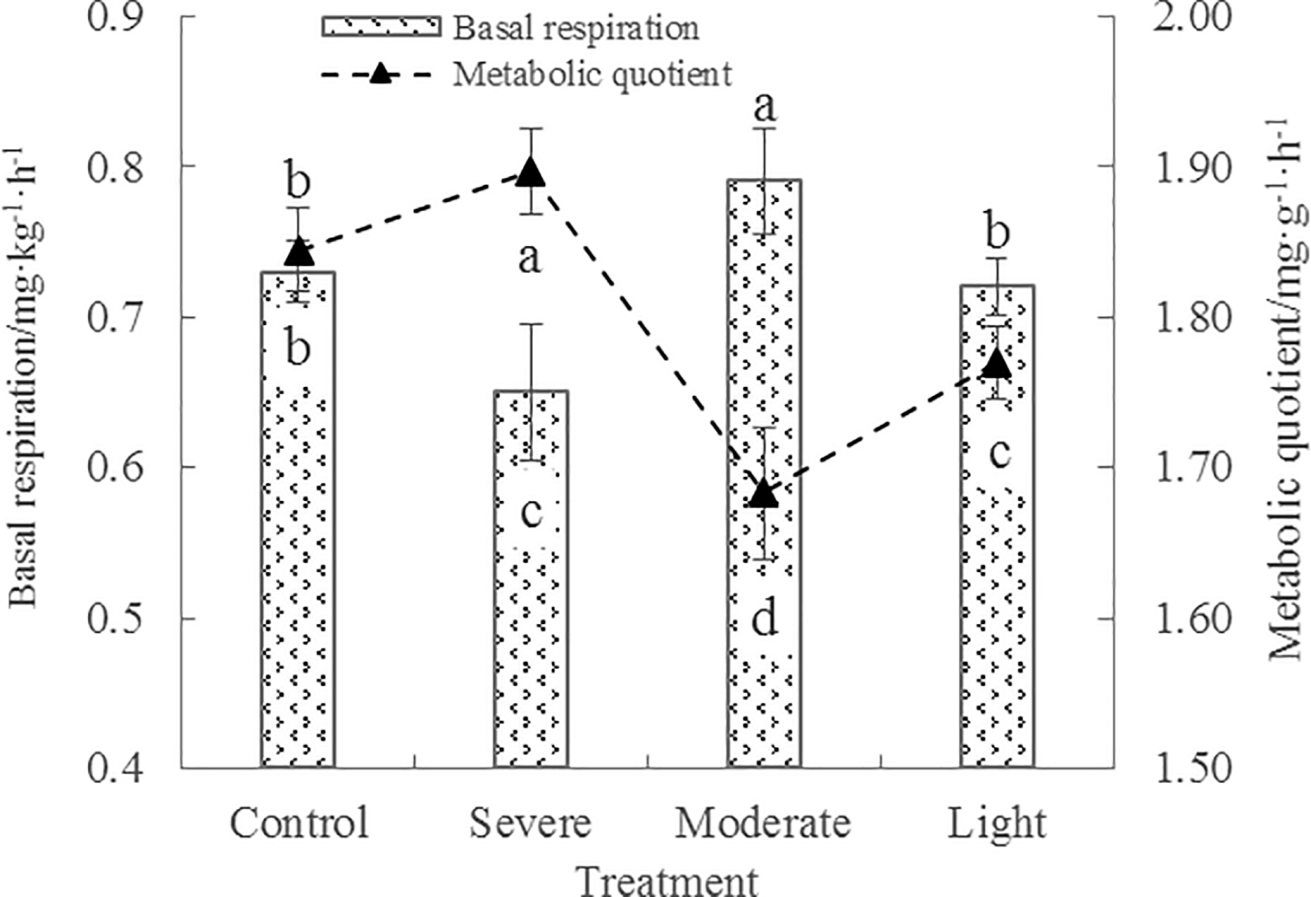
Effects of root pruning on basal respiration and metabolic quotient in the rhizosphere soil of poplar. Bars are means, and error bars are standard deviations (n = 3). Different letters indicate significant differences among treatments at *P*<0.05 by LSD.

**Fig 4:**
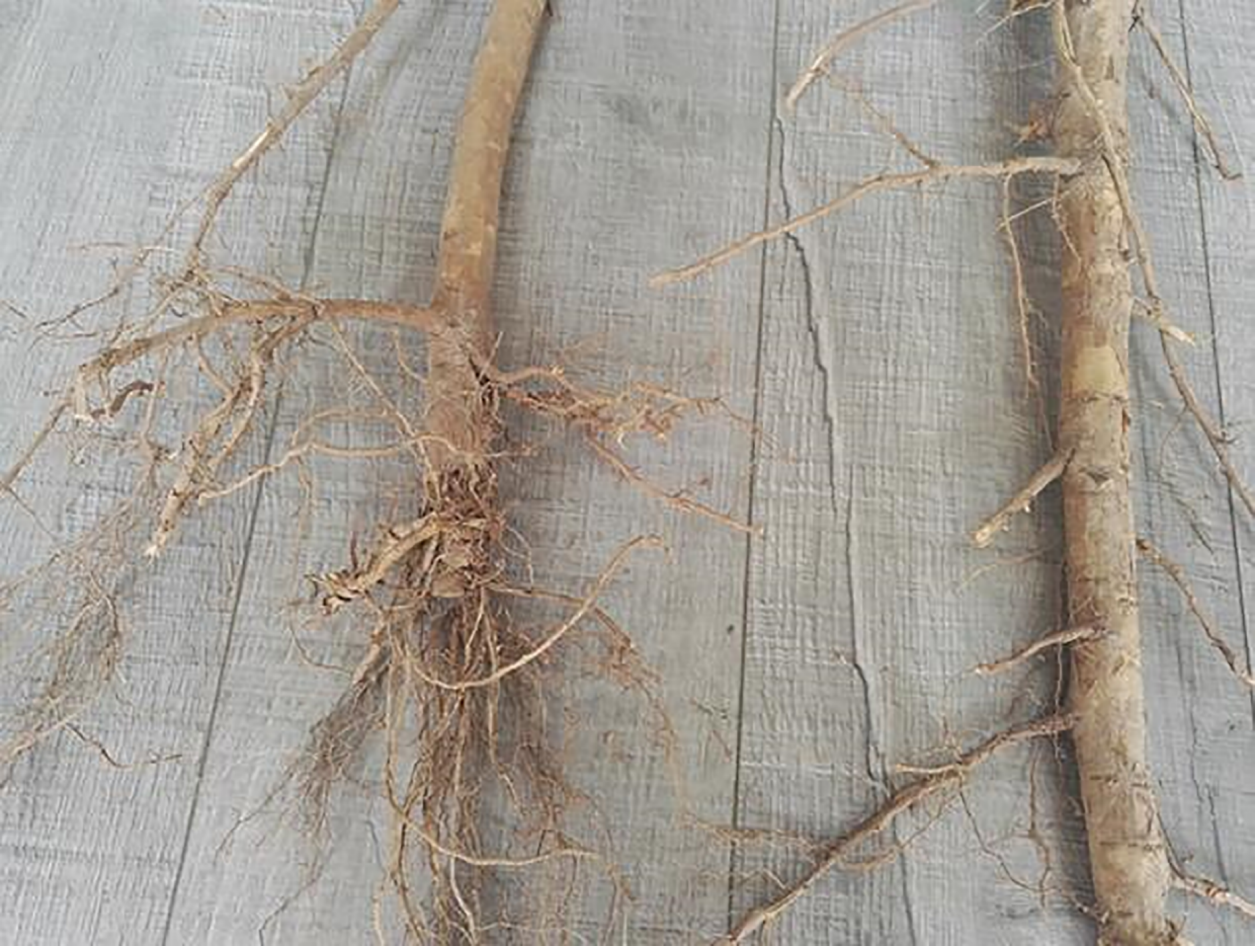
Morphological changes of roots as affected by severe root pruning and control.

Data were analyzed using a completely randomized design. Analysis of variance (ANOVA) was carried out to evaluate the effects of root pruning on the physicochemical properties and microbial activities in the rhizosphere soil and poplar growth. When significant differences were found among the treatments, least significant difference (LSD) test was conducted to detect differences among individual treatment means. All statistical analyses were performed at a significant level of *p* < 0.05. ANOVA and multiple comparisons were performed using SPSS software (version 19.0; SPSS Inc., Chicago, Illinois). Results are presented as means of triplicate measurements and shown in Figs 1–4 and Tables 1–5.

**Table 3:** Effects of root pruning on the root exudates contents in the rhizosphere soil of poplar (mean±SD).

Treatment	Organic acid/(μg·kg^-1^)	Amino acid/(mg·kg^-1^)	Total sugar/(mg·kg^-1^)
**Control**	19.59 ±1.40b	1.38 ± 0.12b	13.76 ± 1.64b
**Severe**	16.67 ± 0.90c	0.75 ± 0.10c	12.89 ± 1.09b
**Moderate**	25.82 ± 1.12a	1.63 ± 0.15a	18.25 ± 0.81a
**Light**	19.06 ± 0.64b	1.29 ± 0.10b	17.93 ± 1.06a

Note: Different letters indicate significant differences among treatments at *P*<0.05 by LSD.

**Table 4:** Effects of root pruning on the physical and chemical properties in the rhizosphere soil of poplar (mean±SD).

Treatment	Available nutrient/(mg·kg^-1^)			pH	Total organic carbon/(g·kg^-1^)	Active organic carbon/(g·kg^-1^)		
	N	P	K			Highly	Moderately	Inertly
**Control**	36.42±2.30b	14.61±0.48b	112.59±9.91b	8.55±0.11b	6.58±0.10b	0.46±0.02c	0.65±0.04c	0.99±0.06c
**Severe**	33.85±1.19c	13.76±0.61c	89.31±9.09c	8.79±0.15a	6.51±0.11b	0.38±0.06d	0.62±0.09c	0.83±0.09d
**Moderate**	47.29±1.87a	18.53±1.07a	137.62±14.48a	8.36±0.06c	6.87±0.09a	0.59±0.04a	0.84±0.06a	1.28±0.09a
**Light**	38.06±1.59b	14.92±0.80b	106.39±16.99b	8.51±0.06b	6.63±0.13b	0.52±0.04b	0.76±0.06b	1.12±0.11b

Note: Different letters indicate significant differences among treatments at *P*<0.05 by LSD.

**Table 5:** Effects of root pruning on the growth rates of DBH, tree height, and volume of poplar (mean±SD).

Treatment	DBH/cm		Tree height/m		Volume (×10^−2^)/m^3^		DBH growth rate/%	Tree height growth rate/%	Volume growth rate/%
	Apri	Nov	Apri	Nov	Apri	Nov			
**Control**	12.29±0.15a	14.37±0.22b	12.72±0.15a	14.68±0.16c	6.33±0.30a	9.99±0.28b	15.60±1.25b	14.31±0.49b	44.83±2.29b
**Severe**	12.43±0.27a	14.18±0.10c	12.65±0.25a	14.21±0.18c	6.44±0.26a	9.42±0.35c	13.15±0.50c	11.62±0.73d	37.52±1.40c
**Moderate**	12.37±0.35a	14.89±0.28a	12.57±0.37a	14.95±0.23a	6.34±0.48a	10.93±0.21a	18.49±0.66a	17.30±0.95a	53.12±1.32a
**Light**	12.52±0.30a	14.56±0.16b	12.79±0.09a	14.56±0.31b	6.61±0.26a	10.18±0.40b	15.07±1.13b	12.94±0.85c	42.49±1.99b

Note: Different letters indicate significant differences among treatments at *P*<0.05 by LSD.

## Results

### Root exudates

Root exudates are important in absorption of nutrients and regulation of environments in rhizosphere soil [14]. Table 3 shows the effects of different root pruning treatments on the contents of organic acid, amino acids, and total sugar in the exudates of poplar rhizosphere soil. Samples treated with moderate root pruning contained the highest amounts of organic acids and amino acids, with levels higher than those in the other treatments. In particular, the organic acid content in the samples treated with moderate root pruning increased by 31.8%, 35.5%, and 54.9%, respectively, compared with the control, light, and severe root-pruning groups. Moreover, the organic acid content in samples treated with light root pruning showed no statistical significant difference with the control group but was significantly higher than that in samples treated with severe root pruning. The total sugar content did not differ significantly between the moderate and light root-pruning treatments but was higher than in either the control or severe root-pruning groups. ANOVA results indicated that different root pruning treatments significantly influenced the contents of root exudates, and the distance from the trunk by root pruning exhibited decisive effects.

### Physicochemical properties of rhizosphere soil

In contrast to the control group, the pH of soil samples treated with light root pruning showed no significant change, whereas the pH of soils treated with moderate root pruning decreased and that of soils treated with severe root pruning significantly increased (Table 4). The contents of available N, P, and K in samples subjected to different root-pruning treatments indicated a similar pattern in variation of moderate > light = control > severe. The contents of available N, P, and K in samples treated with moderate root pruning were higher than those in the other treatments and increased by 29.8%, 26.8%, and 22.2%, respectively, in comparison to the control group. The amounts of these elements in soils in the light root-pruning group were not significantly different from those in the control group, but were significantly higher than those of samples treated with severe root pruning. As shown in Table 4, the TOC in samples treated with moderate root pruning was significantly higher than in the control, light, or severe-pruning groups, whose TOC contents did not differ significantly from one another. Moreover, different root-pruning treatments had unique effects on the three types of active organic carbon. The contents of high, moderate, and inert-activity organic carbon in soils from the moderate root-pruning treatment group were evidently higher than those in the other treatment groups. The amount of these carbon types in soils from the light root-pruning treatment groups increased significantly compared with those in soils from the control and severe root-pruning treatment groups. In addition, the change amplitude of active organic carbon content was larger than that of TOC. Therefore, different distances from the trunk by root pruning exhibited varied effects on the micro-ecological environment of rhizosphere; of the treatments examined in this study, moderate root pruning resulted in the most improved effects.

### MBC, MBN, and microbial quotient

Soil MBC decomposes faster than soil organic matter and plays an important role in soil organic matter decomposition and nutrient transformation [32]. Soil MBN comprehensively reflects the nitrogen mineralization and immobilization effect of the soil microbial community [33]. As shown in Fig 1, the changes in MBC and MBN were similar under each treatment regimen. MBC and MBN in soil samples treated with moderate root pruning were significantly higher than those in the other treatments and increased by 18.6% and 24.3%, respectively in comparison with the control group. MBC and MBN did not differ significantly between the light root pruning and control groups, but were higher than the severe root-pruning group. The MBC: MBN ratios in control, light, moderate, and severe root pruning treatments were 7.04, 7.13, 6.72, and 6.98, respectively. Variance analysis showed that the MBC: MBN ratios did not differ significantly among the control, light, and severe root-pruning treatments, but were significantly higher than in moderate root-pruning treatment. Additionally, *q*MIC ranged from 5.24%–6.83% across the groups, peaking in the moderate root-pruning treatment, where it differed significantly from that in the other treatments (Fig 2). Overall, the moderate root-pruning treatment significantly increased MBC, MBN, and *q*MIC in poplar rhizosphere soil and significantly decreased the MBC: MBN ratio. Thus, the moderate root-pruning treatment promoted changes in the microbial community structure of the soil.

### Basal respiration and metabolic quotient

Fig 3 shows the effects of different root-pruning treatments on the BR of poplar rhizosphere soil. BR in samples treated with moderate root pruning was significantly higher than that of the other treatment groups, showing increases of 8.2%, 9.7, and 21.5% compared with samples treated with control, light, and severe root pruning, respectively. BR did not differ significantly between the light root-pruning and control groups, but was significantly higher than in samples treated with severe root pruning.

*q*CO_2_ refers to the ratio between the soil BR and MBC [34]. This parameter not only represents the size and activity of microbial biomass, but also indicates the maturity of the soil ecosystem [35]. As shown in Fig 3, different root pruning treatments exhibited varied effects on *q*CO_2_. Compared with the control, *q*CO_2_ increased in the severe root-pruning treatment and significantly decreased in the light and moderate root-pruning treatments. The *q*CO_2_ in the moderate root-pruning treatment decreased by 8.9%, 5.0%, and 11.4% compared with the treatments of control, light, and severe root pruning, respectively. Therefore, moderate root pruning not only significantly enhanced the BR of poplar rhizosphere soil, but also decreased *q*CO_2_, thereby maintaining the sustainable utilization potential of the soil.

### Growth rate

Table 5 shows the effects of different root pruning treatments on the growth rates of DBH, tree height, and volume of poplar. The growth rates of DBH, tree height, and volume in moderate root-pruning group significantly increased in contrast to the other treatments. In particular, the volume growth rate in the moderate root-pruning group increased by 18.5%, 25.0%, and 41.6% compared with the treatments of control, light, and severe root pruning, respectively. The growth rates of DBH and volume in samples treated with light root pruning were not significantly different from those of the control, whereas the tree height growth rate was significantly lower than that of the control. The growth rates of DBH, tree height, and volume in samples treated with severe root pruning were significantly lower than those of the other treatments. Therefore, different root pruning treatments displayed varied effects on the growth of poplar; specifically, moderate root pruning led to significantly higher promotion effects compared with the other treatments.

## Discussion

### Physical and chemical properties

**Fig 5:**
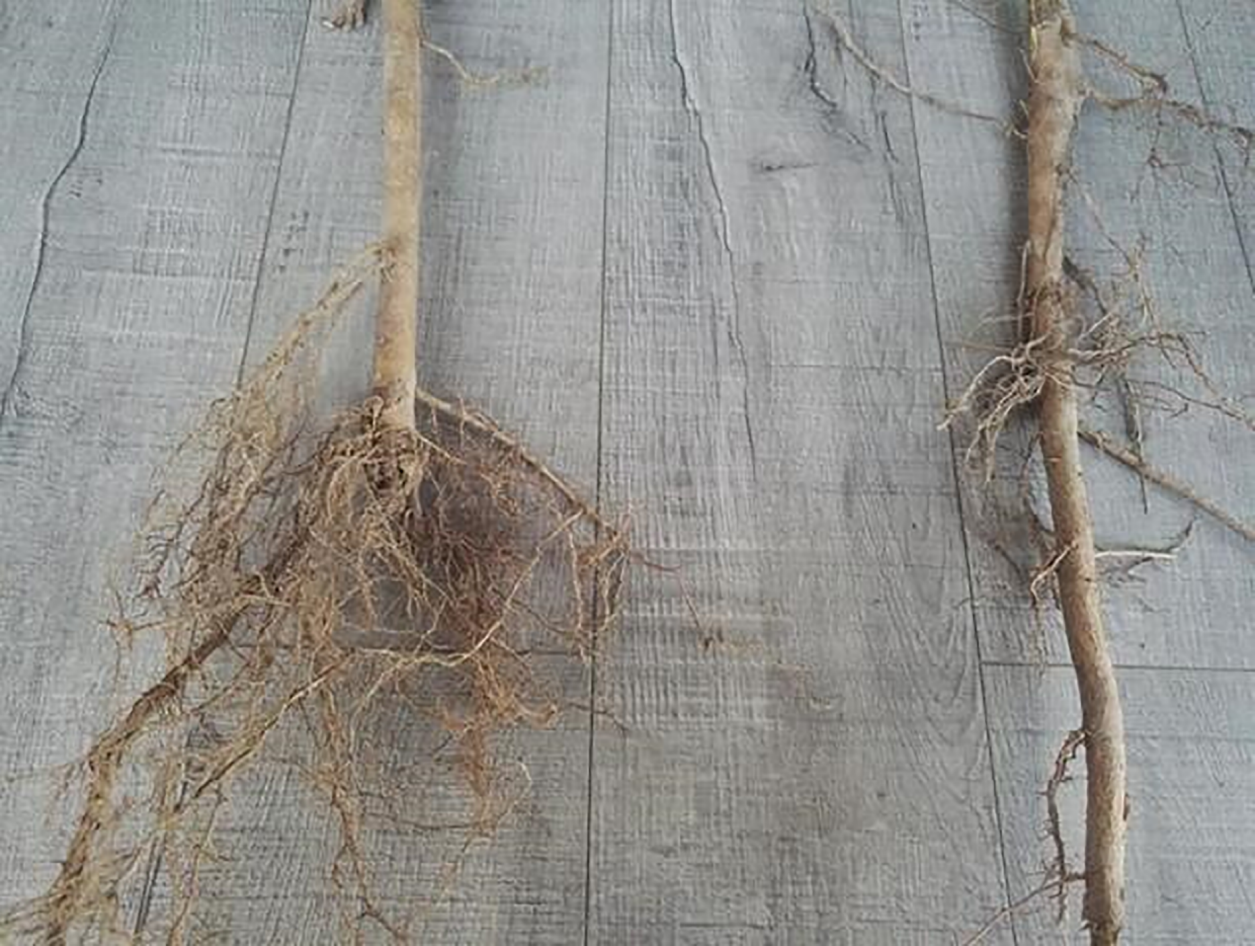
Morphological changes of roots as affected by moderate root pruning and control.

**Fig 6:**
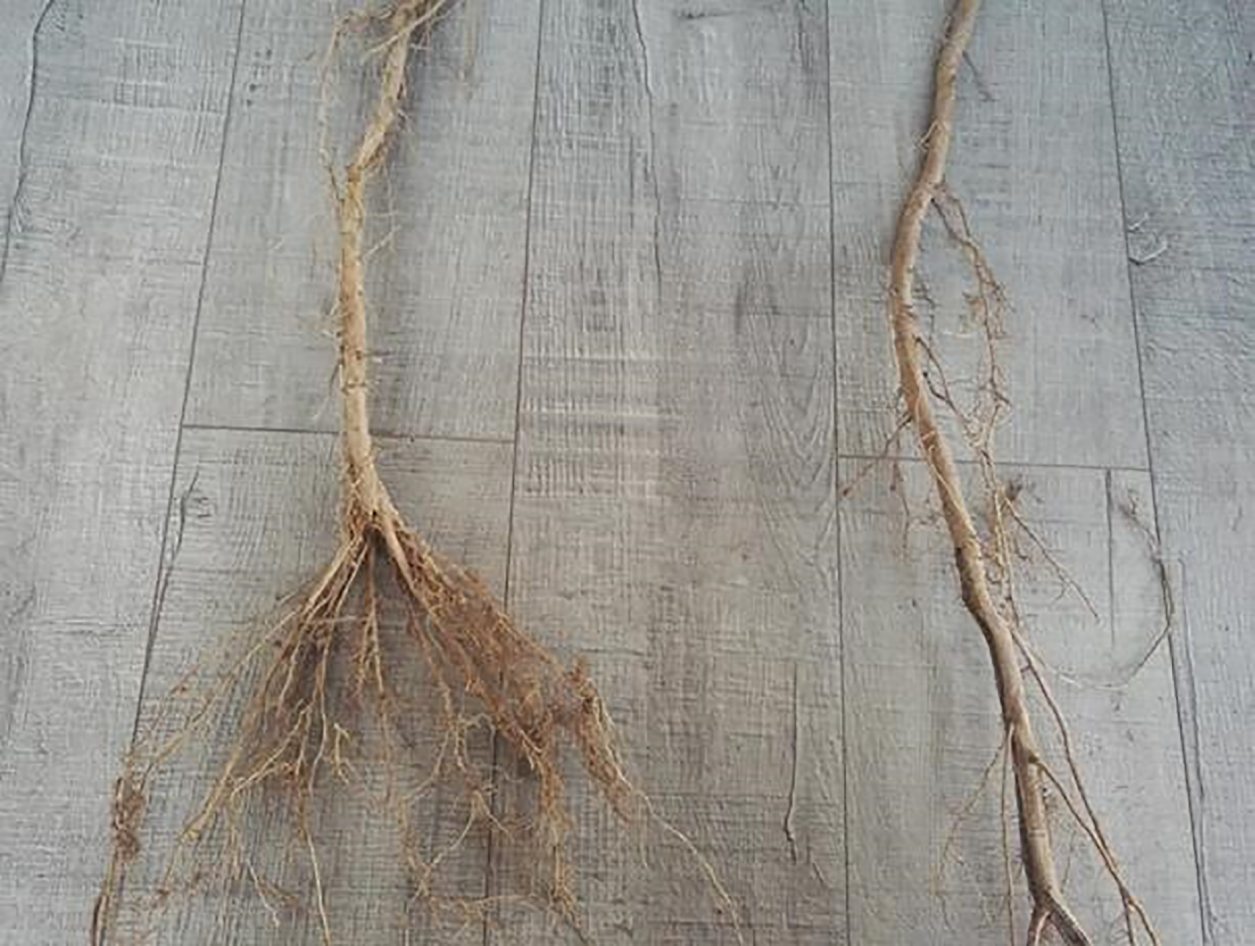
Morphological changes of roots as affected by light root pruning and control.

*Populus* × *euramericana* cv. ‘Neva’ is regarded as one of the most suitable species for afforestation and timber production in arid and semi-arid regions of China. The successful establishment and rapid growth of the trees depend on the fast growth and prominent nutrition absorption capacity of their root systems. In the current study, a portion of lateral roots (rather than fine roots) was cut via root pruning over a period of seven months, which destroyed the original growth environment of the roots and altered the correlation between the roots and the surrounding soil. The lateral root is thicker with fewer hair roots compared with the finer roots and its main function is nutrient transportation and conduction. Before pruning, the physicochemical properties and microbial activities of the rhizosphere soil collected from the points of six times, eight times and ten times the DBH from the trunk were determined (Table 2). The results indicated that there were no significant spatial differences among the different samples collected from 30 trees in each plot. Therefore, we paid particular attention to the effects among the different pruning treatments. Some scholars reported that the disturbances by root pruning played an important role in processing and retention of nutrients in soil-plant system [6–7]. The experimental results showed that moderate root pruning had more positive effects on the physicochemical characteristics of poplar rhizosphere soil compared with the other treatments. During the period of plant growth, the root system not only absorbs nutrients and water from the soil, but also secretes protons, releases inorganic ions, and generates abundant organic matter, which is added to the growth medium (referred to as ‘root exudates’), and have immediate and longer-term effects on nutrient cycling and organic matter accumulation in soil-plant system [15]. Moderate root pruning increased the organic acid content in the root exudates, which decreased the pH of the rhizosphere soil. This decrease in pH improved the solubility of phosphorus, potassium, and some salt ions [14], thereby increasing the contents of available phosphorus and potassium in the rhizosphere soil. This effect could also increase the concentration of nutrient ions in the rhizosphere soil, enhancing soil fertility and facilitating the absorption and utilization of nutrients by the root systems of poplar [4, 6]. Previous studies on apple [36] and wheat [37] reported similar conclusions. Moreover, the present study concluded that moderate root pruning can significantly increase the TOC and active organic carbon (with three different levels of activities) in the rhizosphere soil compared with the control group. This result is likely to be because poplar trees deliver most of their assimilation to the root incision to recover the absorption ability and growth of the root system immediately after root pruning [38]. As such, large amounts of organic matter are found at root incisions [14]. Furthermore, the moderate root-pruning treatment not only increased the content of TOC, but also enhanced the quality of the organic carbon, showing an essential role in the functioning and productivity of forestry systems. The levels of active organic carbon varied more across the different treatment groups compared with TOC, suggesting that active organic carbon elicited a more sensitive reaction to root pruning compared with TOC. In contrast to the control group, light root pruning had no significant effects on the ecological environment of poplar rhizosphere soil; an effect that was significant as a result of moderate root pruning but reversed by severe root pruning. This was mainly because moderate root pruning promoted the germination of a large number of fine roots from the incision (Figs 4–6), thereby significantly enhancing root activity and producing abundant root exudates [4]. Light root pruning had a limited stimulation effect on the incision because of the large distance between the point of root pruning and the trunk; by contrast, severe root pruning severely damaged the root system because of the short distance between the point of pruning and the trunk, delaying the recovery of the root system [7]. Thus, the distance from the trunk by root pruning is important in improving the physicochemical properties of poplar rhizosphere soil.

### MBC, MBN, and microbial quotient

Microbial biomass can be a sensitive indicator of changes in soil processes because of its faster turnover rate than total soil organic matter [39–40]. Some scholars reported a strong correlation among soil microbial biomass, soil fertility, and soil quality [41–42].

Soil MBC and MBN are important parameters that reflect the cycling and transformation of soil carbon and nitrogen as well as the microbial growth state and fertility of the soil [32, 43]. Root pruning caused the disturbances to the soil-plant system and therefore induced the changes in microbial biomass in the rhizosphere solution [14]. Du et al. [4] reported that moderate root pruning increased the microbial population in rhizosphere soil, which had a beneficial effect on MBC and MBN. This experimental result was also observed in our study. This result is likely because moderate root pruning improved the ecological environment of the rhizosphere soil, thereby creating a favorable environment for microbial growth and promoting the mass propagation of soil microbes [4]. The MBC: MBN ratio is linked to the microbial community structure, with lower values generally indicating a shift from fungi to bacteria [43–44]. Hence, the lower MBC: MBN value observed in the moderate root-pruning group could be related to a possible shift from fungi to bacteria in the rhizosphere soil. Moreover, *q*MIC (MBC/TOC) is considered to be a more sensitive indicator of soil fertility than either MBC or TOC alone [45–46]. In the present study, higher MBC/TOC values were observed in the moderate root-pruning groups, with a positive effect on soil fertility.

### Basal respiration and metabolic quotient

Soil BR is widely used as a measure of microbiological activity and as a sensitive indicator of microbial activity [47–48]. In the current study, moderate root pruning significantly increased the BR of rhizosphere soil. On the one hand, moderate root pruning significantly increased the content of readily decomposable organic matter in the rhizosphere soil and the soil respiration substrates [4]. On the other hand, improvements in the physicochemical properties of the rhizosphere soil enhanced the vitality of soil microbes [14]. Hence, moderate root pruning not only increased soil microbial biomass but also improved the potential mineralization rate of organic matter, resulting in enhanced overall activities of the soil microbial community.

*q*CO_2_ organically links the size of the microbial biomass with the biological activity and function of the microbial community [32, 34]. This parameter can reveal the physiological characteristics of a microbial community [21, 33]. Previous studies reported that low *q*CO_2_ indicates the high carbon utilization efficiency of a soil microbial community [34, 49]. By contrast, high *q*CO_2_ indicates that a relatively low proportion of carbon is being used for microbial cell synthesis and, therefore, there is a low utilization efficiency of the carbon source [35]. In this study, the *q*CO_2_ was significantly increased by severe root pruning but was significantly decreased by light and moderate root pruning. In particular, *q*CO_2_ was lower in samples treated with moderate root pruning than in the other treatments. Similar trends in *q*CO_2_ have also been reported by other authors [43, 50]. On the one hand, although the BR and MBC in samples treated with moderate root pruning showed consistent change, MBC increased rapidly and *q*CO_2_ decreased. On the other hand, this treatment promoted the mass propagation of microbes and increased microbial activities, leading to a significant increase in microbial biomass [51–52]. Therefore, the utilization efficiency of the carbon source was improved [34]. Furthermore, root pruning inevitably disturbs the old growth balances of trees and alters their assimilation abilities, nutrient distributions and the root environment, resulting in different effects on growth. In this study, we found that the growth rates of DBH, tree height, and volume of poplar treated with pruning at eight times the DBH from the trunk were significantly higher than those of the other treatments. It is likely that the poplar trees were able to absorb adequate nutrients and water for the growth of aboveground organs because their existing root surface area was increased as a result of pruning (Figs 4–6). In addition, this root-pruning treatment significantly improved the physicochemical properties and microbial activity of rhizosphere soil, enhancing the micro-ecological environment of the root system, and resulting in improved soil fertility and growth of the trees. Moreover, the microbial biomass appears to be closely linked to aboveground plant productivity in many ecosystems [53]. Therefore, improvements in the physicochemical properties of, and microbial activities in, the rhizosphere soil also promoted the growth of the trees. Thus, the distance from the trunk by root pruning is vital to improve the physicochemical properties of, and microbial activities in, poplar rhizosphere soil. However, the effects observed in the current study might also be related to soil characteristics, planting densities and stand age.

## Conclusions

The results of this study indicate that the application of root pruning is an effective way to improve the physicochemical properties of, and microbial activities in, rhizosphere soil and enhance poplar growth at the canopy-closing stage. Root pruning, especially the moderate pruning is beneficial to improving the physicochemical properties and microbial activities of rhizosphere soil, and increasing the growth rates of DBH, tree height and volume. Therefore, the selection of the distance from the trunk by root pruning plays a decisive role in the effectiveness of this technique for the management of poplar plantations. Hence, root pruning can ameliorate the physicochemical characteristics and improve the growth of poplar, showing a real potential to perform as a promotion measure in the cultivation of poplar plantation.

## Supporting information

S1 File Morphological changes of roots as affected by different pruning treatments. (DOCX)

S2 File The physicochemical properties and microbial activities from the points of six times, eight times and ten times the DBH from the trunk of poplar before pruning (mean±SD). (XLSX)
