# Supplementary material for: Effects of root pruning on the physicochemical properties and microbial activities of poplar rhizosphere soil
Source: PLoS One. 2017 Nov 8;12(11):e0187685. doi: 10.1371/journal.pone.0187685 (PMC5678864; doi:10.1371/journal.pone.0187685)
Supplement: S1 File — (DOCX) [file pone.0187685.s001.docx]

**S1 File. Morphological changes of roots as affected by different pruning intensities.**


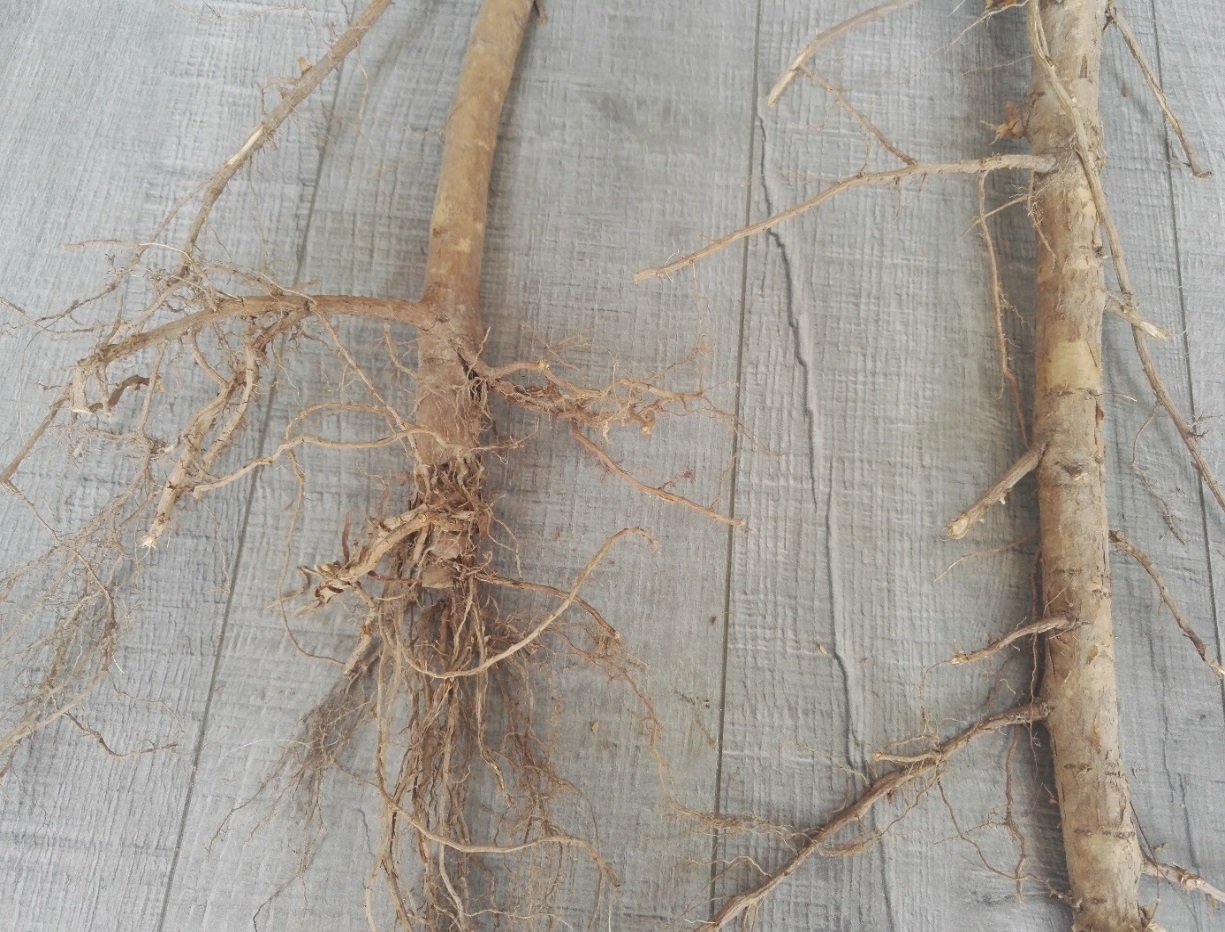


(a) severe root pruning and control


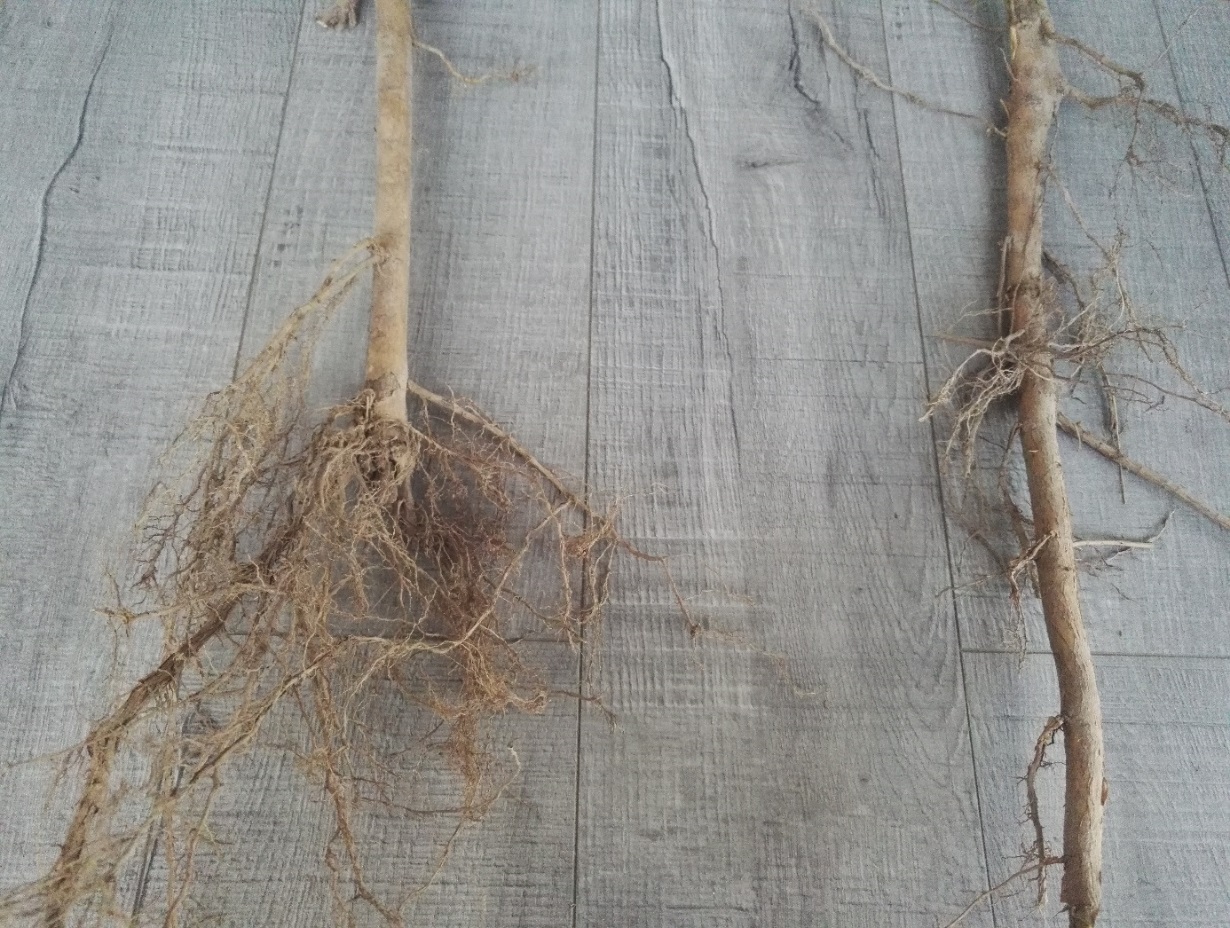


(b) moderate root pruning and control


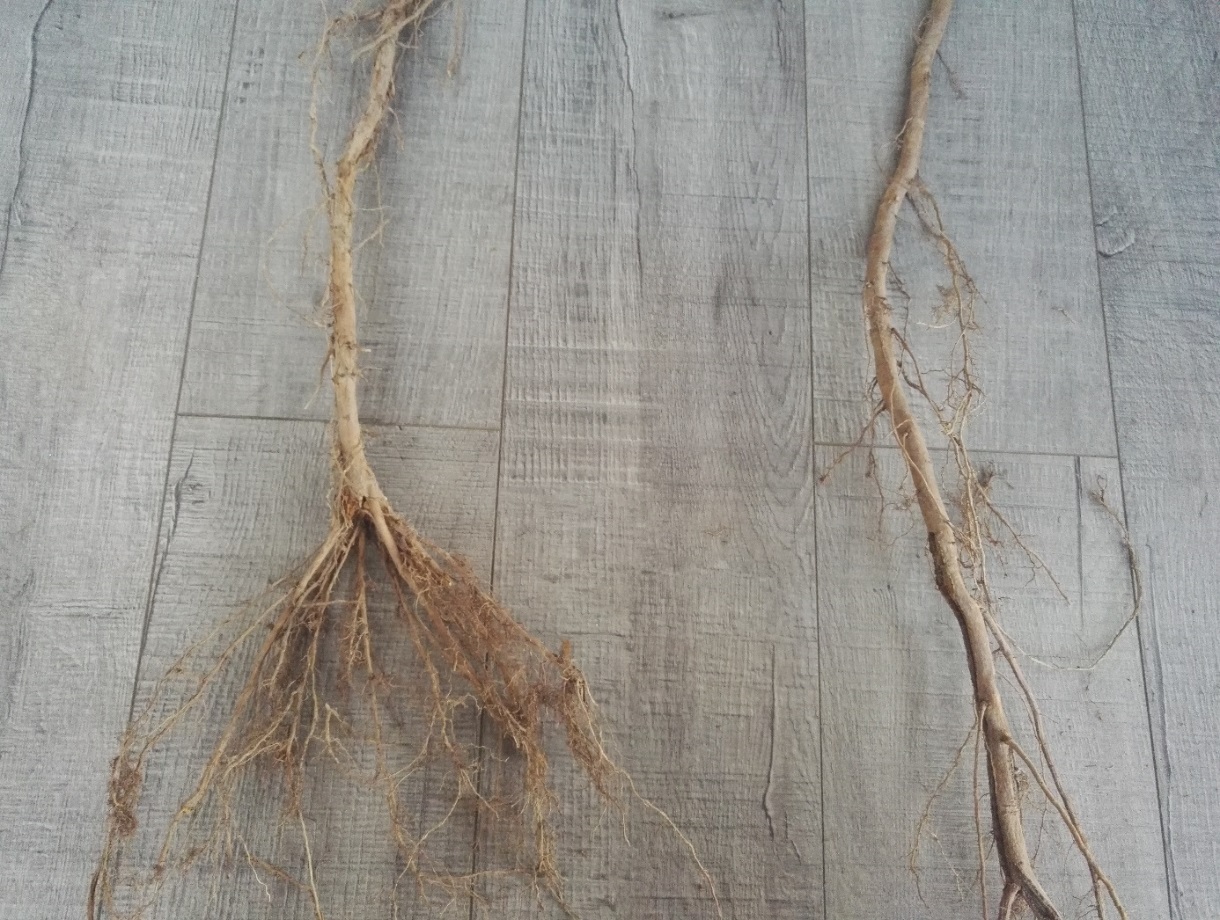


(c) light root pruning and control
